# Evaluating the Transient Energy Dissipation in a Centrifugal Impeller under Rotor-Stator Interaction

**DOI:** 10.3390/e21030271

**Published:** 2019-03-11

**Authors:** Ran Tao, Xiaoran Zhao, Zhengwei Wang

**Affiliations:** Department of Energy and Power Engineering, Tsinghua University, Beijing 100084, China

**Keywords:** centrifugal impeller, rotor-stator interaction, energy dissipation, entropy production, flow-induced noise

## Abstract

In fluid machineries, the flow energy dissipates by transforming into internal energy which performs as the temperature changes. The flow-induced noise is another form that flow energy turns into. These energy dissipations are related to the local flow regime but this is not quantitatively clear. In turbomachineries, the flow regime becomes pulsating and much more complex due to rotor-stator interaction. To quantitatively understand the energy dissipations during rotor-stator interaction, the centrifugal air pump with a vaned diffuser is studied based on total energy modeling, turbulence modeling and acoustic analogy method. The numerical method is verified based on experimental data and applied to further simulation and analysis. The diffuser blade leading-edge site is under the influence of impeller trailing-edge wake. The diffuser channel flow is found periodically fluctuating with separations from the blade convex side. Stall vortex is found on the diffuser blade trailing-edge near outlet. High energy loss coefficient sites are found in the undesirable flow regions above. Flow-induced noise is also high in these sites except in the stall vortex. Frequency analyses show that the impeller blade frequency dominates in the diffuser channel flow except in the outlet stall vortexes. These stall vortices keep their own stall frequency which is about 1/5 impeller frequency with high energy loss coefficient but low noise level. Results comparatively prove the energy dissipation mechanism in the centrifugal air pump under rotor-stator interaction. Results also provide the quantitative basis for turbomachinery’s loss reduction design.

## 1. Introduction

Energy conversion is a key issue in turbomachinery flow cases. According to the first law of thermodynamics, the total energy of an isolated system is constant. The energy can be transformed from one system to another system but can be neither created nor destroyed. Thus, the energy conversion including heat transfer, mass transfer and force working finally performs as the total energy change [1]. Energy in flowing fluid includes the internal energy, potential energy, kinetic energy and the product of its pressure and volume [2]. In turbomachinery, if the heat exchange between medium and turbomachine body and the mass transfer are ignored, the mechanical energy loss of fluid medium should be equal to the increasing of thermodynamic energy which is the product of the specific heat capacity and temperature [3]. Briefly speaking, the performance of turbomachinery performs as the temperature changes.

In the actual studies of the performance of aerodynamic and hydraulic flow cases, checking the energy dissipation can be helpful to understand the system energy loss. Entropy, which represents the system chaos, were introduced to describe the irreversibility of thermodynamic system [4]. Diaconescu [5] analyzed the heat-insulated flow in pipelines. The relationship between entropy change and pressure drop was discussed in detail. Esfahani et al. [6] also studied the entropy production in heat-insulated flow in pipelines. The numerical results were verified by comparison with the experimental data. Yoon et al. [7] analyzed the entropy production in different parts of a turbine stage. The energy losses were quantitatively studied and clarified for a better understand. Jia et al. [8] simulated the flow in a highly-loaded turbine. The analysis of entropy production also helped the judgement of energy loss. VanZante et al. [9] used the concept of irreversibility to understand the loss in compressor. Liu et al. [10] simulated and analyzed the irreversibility represented hydraulic loss in a shroudless hydro turbine. Sun et al. [11] also studied the irreversibility in a compressor in different region like tip leakage, wake and vaneless region. Li et al. [12] numerically and experimentally discussed the energy loss in the reversible pump-turbine based on entropy production analysis. These studies above find the energy loss site and intensity. Understanding the energy dissipation would also help the design of turbomachinery by reducing the loss. Kluxen et al. [13] studied the entropy production due to backflow in axial-flow turbine. Soltanmohamadi et al. [14] optimized the turbine based on entropy production analysis. The energy loss was strongly reduced by over 25% in a wide operation range. Zeinalpour et al. [15] introduced the optimization strategy based on the continuous adjoint formulation for designing the turbine cascade. These studies show that entropy production analysis has great meaning in guiding the optimization design of turbomachinery.

Generally, one can connect the flow regime with the energy dissipation through computational fluid dynamics (CFD) simulation methods. The entropy production strongly relates to the undesirable flow structures. However, the entropy production intensity is not clear under strongly transient flow situation especially in the rotating turbomachinery with rotor and stator. Rotor-stator interaction, as a complex issue in turbomachinery flow cases, has received typical studies in the past by both numerical simulation and experiment [16,17,18]. In this study, the typical centrifugal air pump flow case is studied by CFD simulation under a reduced rotating speed. The transient flow regime and energy loss are comparatively studied by monitoring the velocity vectors, turbulence kinetic energy fields and specific entropy fields. The field of flow-induced noise is also discussed based on the acoustic analogy method to approximately know the energy transition to acoustic energy [19,20]. This study will give a reference to evaluate the energy dissipation in turbomachinery especially considering the rotor-stator interactions.

## 2. Numerical Methods

### 2.1. Governing Equations

In this low Mach number air pump case, the fluid medium is treated as incompressible. The thermodynamic effects are taken into consideration in this 3D incompressible viscous turbulent flow. The time-averaged equations [21], known as Reynolds-averaged Navier-Stokes (RANS) equations, are used to decompose the quantities into time-averaged component and fluctuating component. Thus, the continuity equation and momentum equation can be written as:

(1) $$\frac{\partial \bar{u_{i}}}{\partial x_{i}}=0$$

(2) $$\rho \frac{\partial \bar{u_{i}}}{\partial t}+\rho \bar{u_{j}}\frac{\partial \bar{u_{i}}}{\partial x_{j}}=\frac{\partial }{\partial x_{j}}\left(-\bar{p}\delta_{ij}+2\mu \bar{S_{ij}}-\rho \bar{u_{i}^{\prime }u_{j}^{\prime }}\right)$$

where *u* is velocity, *t* is time, *ρ* is density, *x* is coordinate component, *δ_ij_* is the is the Kroneker delta, *μ* is dynamic viscosity. $\bar{\phi }$ and ϕ′ are respectively the time-averaged and fluctuating component of arbitrary parameter ϕ. Term *ρ*$\bar{u_{i}^{\prime }u_{j}^{\prime }}$ is called the Reynolds stress. $\bar{S_{ij}}$ is the mean rate of strain tensor:

(3) $$\bar{S_{ij}}=\frac{1}{2}\left(\frac{\partial \bar{u_{i}}}{\partial x_{j}}+\frac{\partial \bar{u_{j}}}{\partial x_{i}}\right)$$

The total energy equation can be written as:

(4) $$\frac{\partial }{\partial t}\left(\rho h_{tot}\right)-\frac{\partial p}{\partial t}+\frac{\partial }{\partial x_{j}}\left(\rho u_{j}h_{tot}\right)=\frac{\partial }{\partial x_{j}}\left(\lambda_{t}\frac{\partial T}{\partial x_{j}}-\bar{u_{j}h_{sta}}\right)+\frac{\partial }{\partial x_{j}}\left[u_{j}\left(2\mu \bar{S_{ij}}-\rho \bar{u_{i}^{\prime }u_{j}^{\prime }}\right)\right]$$

where *T* is temperature, *h_sta_* is the static enthalpy, *h_tot_* is the total enthalpy that *h_tot_*=*h_sta_*+*u*^2^/2, *λ_t_* is the thermal conductivity. Based on these equations above, the hydraulic energy loss, dissipation and transformation to internal energy can be simulated in detail.

### 2.2. Eddy Viscosity Turbulence Modeling

The RANS equations are not closed after the quantity-decomposition. Therefore, the concept of eddy viscosity is introduced [22] by establishing the relationship between the Reynolds stress *ρ*$\bar{u_{i}^{\prime }u_{j}^{\prime }}$ and the eddy viscosity *μ_t_* as:

(5) $$-\rho \bar{u_{i}^{\prime }u_{j}^{\prime }}=2\mu_{t}\bar{S_{ij}}-\frac{2}{3}k\delta_{ij}$$

where *k* is the turbulence kinetic energy. Thus, eddy viscosity turbulence models can be built to close the RANS equations by modeling the eddy viscosity *μ_t_* based on statistics or experimental verifications.

In this study, the SST (shear stress transport) model which hybrids the standard *k-ε* model and Wilcox *k-ω* model is applied [23,24,25]. It has the advantage in treating the high pressure gradient, strong shear flow and the near wall separations. The turbulence kinetic energy *k* equation and specific dissipation rate *ω* equation can be specified as:

(6) $$\frac{\partial \left(\rho k\right)}{\partial t}+\frac{\partial \left(\rho u_{i}k\right)}{\partial x_{i}}=P-\frac{\rho k^{3/2}}{l_{k-\omega }}+\frac{\partial }{\partial x_{i}}\left[\left(\mu +\sigma_{k}\mu_{t}\right)\frac{\partial k}{\partial x_{i}}\right]$$

(7) $$\frac{\partial \left(\rho \omega \right)}{\partial t}+\frac{\partial \left(\rho u_{i}\omega \right)}{\partial x_{i}}=C_{\omega }P-\beta \rho \omega^{2}+\frac{\partial }{\partial x_{i}}\left[\left(\mu_{l}+\sigma_{\omega }\mu_{t}\right)\frac{\partial \omega }{\partial x_{i}}\right]+2\left(1-F_{1}\right)\frac{\rho \sigma_{\omega 2}}{\omega }\frac{\partial k}{\partial x_{i}}\frac{\partial \omega }{\partial x_{i}}$$

where *l_k-ω_* is the turbulence scale which can be expressed as:

(8) $$l_{k-\omega }=k^{1/2}\beta_{k}\omega$$

and *P* is the production term, *C_ω_* is the coefficient of the production term, *F*_1_ is the blending function, *σ_k_*, *σ_ω_* and *β_k_* are model constants.

### 2.3. Acoustic Analogy Method

In this study, the Lighthill acoustic analogy method is also applied based on the turbulent flow modeling. It can apply the near-field of flow-induced noise by predicting sound power level *W_A_* [26]:

(9) $$W_{A}=\alpha_{\varepsilon }\rho \varepsilon M_{t}^{5}$$

where *α_ε_* is a constant equal to 0.1, *ε* is the eddy dissipation rate, *M_t_* is the specific turbulence kinetic energy which can be calculated by:

(10) $$M_{t}=\frac{\sqrt{2k}}{V_{c}}$$

where *V_c_* is the sound speed which is 340 m/s in this case. The flow-induced sound power level *L_sp_* can be calculated by:

(11) $$L_{sp}=10\log_{10}\left(\frac{W_{A}}{W_{ref}}\right)$$

where *W_ref_* is the reference sound power which is 1 × 10^−12^ W/m^3^ in this case.

## 3. Case Description

### 3.1. Centrifugal Air Pump Model

**Figure 1:**
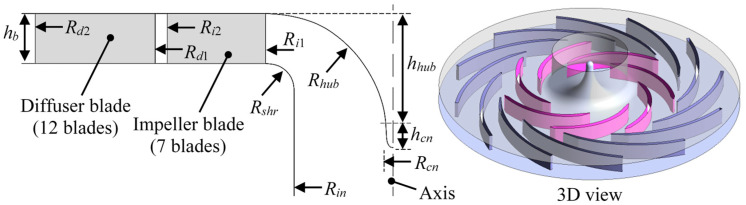
Centrifugal air pump and the indication of its geometric parameters.

The studied centrifugal air pump [27] is shown in Figure 1. It has a 7-bladed radial impeller and a 12-bladed radial diffuser. The value and unit of the geometric parameters are listed in Table 1. The performance parameters and fluid medium properties are listed in Table 2. This is a typical turbomachinery study case which illustrate the centrifugal accelerating flow passing through the rotation impeller and stationary vaned diffuser. Based on the simple blade geometry without twisting, the three-dimensional rotor-stator flow case can be simplified into a quasi-two-dimensional case. Thus, many researchers discussed the flow uniformity, jet-wake structure, leading-edge separation and other typical flow characteristics based on this centrifugal pump model [28,29,30,31]. The numerical methods by solving RANS turbulent flow with SST *k*-*ω* model are also verified based on this typical pump unit. To describe the flow rate condition, the dimensionless flow rate coefficient *C_φ_* can be expressed as:

(12) $$C_{\varphi }=\frac{Q}{\pi U_{i2}R_{i2}^{2}}$$

where *Q* is flow rate.

### 3.2. Flow Domain Meshing

The flow domain including the impeller and diffuser should be discretized (meshed) for CFD simulation. In this study, flow domain is meshed by using the commercial software ICEMCFD. The structural mesh is used with hexahedral elements for both the impeller and the diffuser. Two parameters, the specific entropy difference coefficient Δ*C_stot_* between impeller inlet and diffuser outlet and the *y^+^*, are checked to determine the mesh scheme for a better simulation accuracy. The specific entropy difference coefficient Δ*C_stot_* can be written as:

(13) $$\Delta C_{stot}=\frac{T_{x2}s_{x2}^{*}-T_{x1}s_{x1}^{*}}{2gR_{i2}}$$

where s^*^ is the specific entropy, *g* is the acceleration of gravity, subscript *x*_1_ and *x*_2_ denotes two locations. If *x*_1_ is the impeller inflow and *x*_2_ is the diffuser outflow, Δ*C_stot_*, in the case that temperature changes slightly, can describe the energy loss in the flow passage from impeller inflow to diffuser outflow by considering ΔT·s* instead of ∫Td*s* [32]. Checking Δ*C_stot_* can give a better result in predicting the macro energy change in pump. Checking *y^+^* is to improve the near-wall solution especially in the viscous sub-layer and buffer layer.

**Figure 2:**
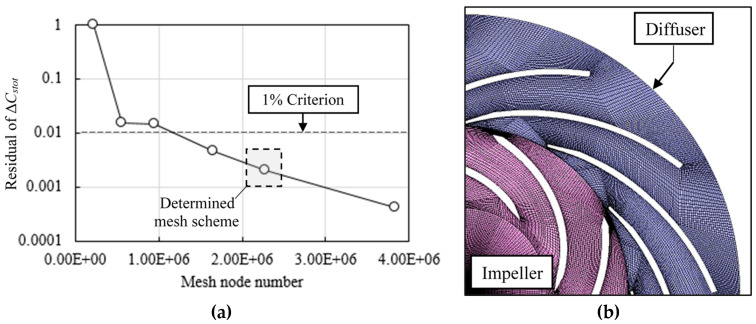
Mesh scheme determination and diagram (**a**) mesh scheme determination by checking the residual of Δ*C_stot_*; (**b**) diagram of the mesh scheme.

In this study, the Δ*C_stot_* check is conducted under varying mesh node number from about 2.23 × 10^5^ to about 3.56 × 10^6^ based on the steady state simulation. The residual of *C_stot_* is monitored as shown in Figure 2 with the criterion of continually less than 1%. The final mesh is determined with 2,271,260 nodes and 2,073,394 elements in total. The *y^+^* value is controlled within 7.8~149.3 on both the impeller blades and diffuser blades by setting the first off-wall layer height of 0.03 mm. This *y^+^* range can be proper for applying the automatic wall treatment [33].

### 3.3. Setup of CFD Simulation

**Figure 3:**
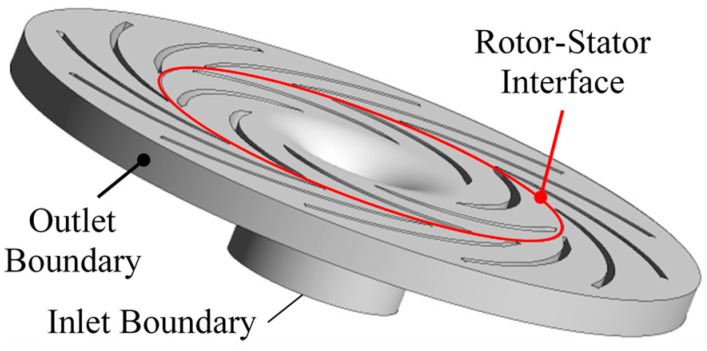
Fluid domain in CFD simulation.

In this case, the internal flow in the centrifugal air pump is solved based on Equations (1) to (8) using the finite volume method based on the commercial software ANSYS CFX. The fluid domain used in CFD simulation is shown in Figure 3 based on the numerical methods in Section 2, the fluid medium property in Section 3.1 and the mesh in Section 3.2. The multiple reference frame model is used so that the impeller domain is rotating by *n* = 1750 r/min and the diffuser domain was stationary. Boundary conditions as partially indicated in Figure 3 includes the following parts:

- Velocity inlet: the velocity at inlet boundary, *V_in_*, was *V_in_* = *Q*/*A_in_* where *A_in_* is the impeller inflow area; the temperature at inlet boundary, *T_in_*, was 298.15 K; the pressure at inlet boundary, *p_in_*, follows the Neumann condition [34]; the inlet turbulence intensity is set as medium of 5%;
- Pressure outlet: the pressure at outlet boundary, *p_out_*, was 0 Pa relative to the environment pressure 1 atm; the velocity and temperature at outlet boundary, *V_out_* and *T_out_*, follows the Neumann condition;
- No slip wall: the impeller hub, impeller shroud, impeller blade, diffuser hub, diffuser shroud and diffuser blade are all in the no slip wall type [35];
- Rotor-Stator interface: an interface is given between impeller and diffuser with conservative interface flux on mass and momentum; the mesh is connected using the general grid interface (GGI) method.

The steady state simulation is conducted as the initial condition. The maximum iteration number is 600 under the criterion of root mean square (RMS) residual less than 1 × 10^−5^. The transient simulation is conducted for about 0.343 s (10 impeller revolutions). The time step is 9.52 × 10^−5^ s with the maximum iteration step number of 10 and the criterion of RMS residual less than 1 × 10^−6^. The advection scheme is set as high resolution.

## 4. Numerical-Experimental Verification Study

**Figure 4:**
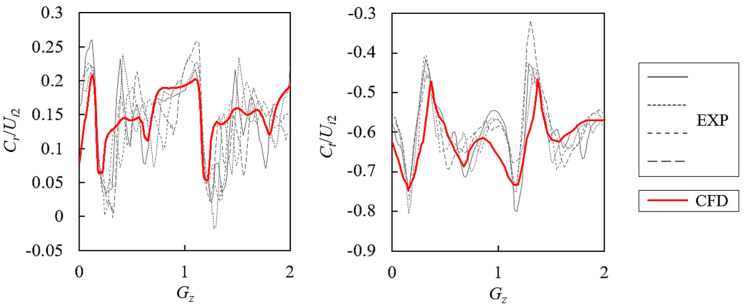
Comparison of the *C_r_*/*U_i_*_2_ and *C_t_*/*U_i_*_2_ on *R*/*R_i_*_2_ = 1.02 and mid-span position within two impeller blade channels. EXP: experimental data at 4 equal-difference acquisition times [27,30]; CFD: numerical data.

**Figure 5:**
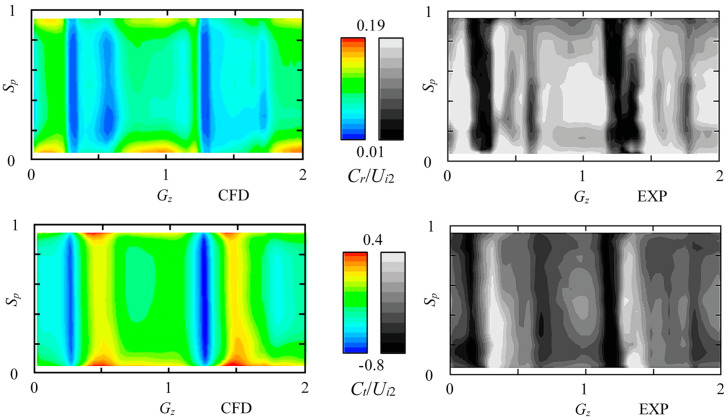
Comparison of the *C_r_*/*U_i_*_2_ and *C_t_*/*U_i_*_2_ on *R*/*R_i_*_2_ = 1.02 circumferential surface within two impeller blade channels. EXP: experimental data [27,30]; CFD: numerical data.

To have a reliable numerical result and a better analysis, the numerical-experimental verification is conducted under the original rotational speed of 2000 r/min. The distribution of velocity against *U_i_*_2_ is chosen for verification by dividing into radial component *C_r_* and tangential component *C_t_*. Figure 4 shows the comparison between experimental and numerical data by plotting curves. Figure 5 shows the comparison between experimental and numerical data on contours. Parameter *G_z_* is the relative impeller blade channel position where 0~2 means two channels. Parameter *S_p_* is the spanwise position where 0 is at hub and 1 is at shroud.

As compared in Figure 4 and Figure 5, the numerical simulation using the RANS method with the SST *k-ω* model can predict the similar pattern to the experimental data [27]. In Figure 4, four equal-difference acquisition times are shown. Both the radial velocity component and the tangential component are within the variation range of experimental data. In Figure 5, the high and low velocity sites accord well with the experimental contour. Flow non-uniformity can be observed between the impeller and vaned diffuser. Generally, the numerical simulation is accurate enough to predict the internal flow field and can be used for the energy loss prediction.

## 5. Transient Flow Field Analysis at Lower-Load

### 5.1. Velocity Fields

**Figure 6:**
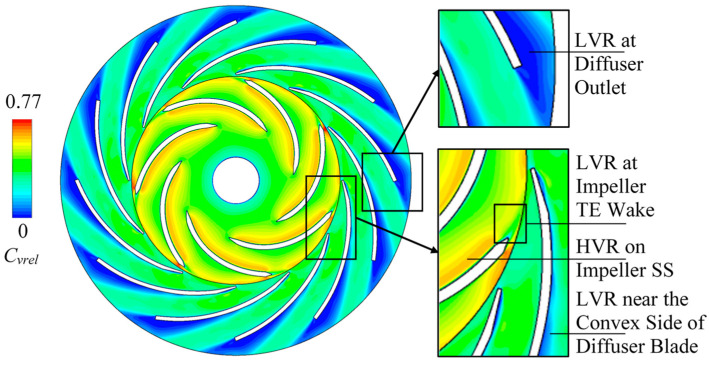
Instantaneous contour of relative velocity coefficient *C_vrel_* with indications of the local low and high regions. LVR: low *C_vrel_* region; HVR: high *C_vrel_* region; SS: blade suction side.

In this study, transient energy loss during rotor-stator interaction is the key issue. Thus, the rotational speed is reduced from original 2000 r/min to 1750 r/min. Based on the reduction, a lower-load situation can be discussed with stronger interaction effects. It can illustrate the transient energy loss happened under the undesirable flow patterns. Based on the settings above, the instantaneous relative velocity contour which indicated the relative flow regime in impeller and diffuser is plotted as shown in Figure 6. The relative velocity coefficient *C_vrel_* is defined as:

(14) $$C_{vrel}=\frac{V_{rel}}{U_{i2}}$$

where *V_rel_* is the relative velocity.

**Figure 7:**
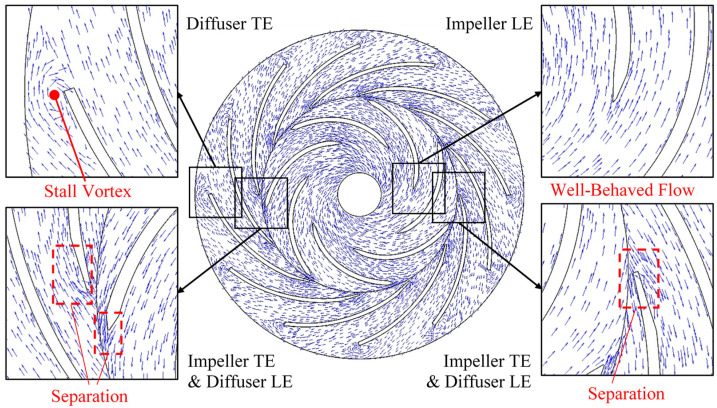
Instantaneous vectors of relative velocity with enlarged views. LE: leading-edge; TE: trailing-edge.

As indicated in Figure 6, high *C_vrel_* region can be mainly found on the suction side of impeller blade and also found near the impeller blade trailing-edge on the pressure side. There are also three mainly low *C_vrel_* regions that located at the diffuser outlet, in the impeller trailing-edge wake and near the convex side of diffuser blade. Figure 7 shows the instantaneous flow regime using relative velocity vectors with enlarged views around leading-edges and trailing-edges. A stall vortex flow can be observed in the diffuser outlet low *C_vrel_* region. Flow separations are also found on the convex side of the diffuser blade near leading-edge and in the impeller blade’s trailing-edge wake. On the contrary, the flow regime is well-behaved near the impeller leading-edge especially on the blade suction side. Therefore, the low *C_vrel_* region is related to the local undesirable flow pattern like separation, wake and vortex. The high *C_vrel_* region is because of the smooth local-flow.

### 5.2. Energy Dissipation Analysis

The energy dissipation in the centrifugal air pump is analyzed based on the CFD simulation. According to Equation (13), the dimensionless energy dissipation coefficient C_stot_^*^ can be defined as:

(15) $$C_{stot}^{*}=\frac{T\cdot s^{*}}{2gR_{i2}}$$

**Figure 8:**
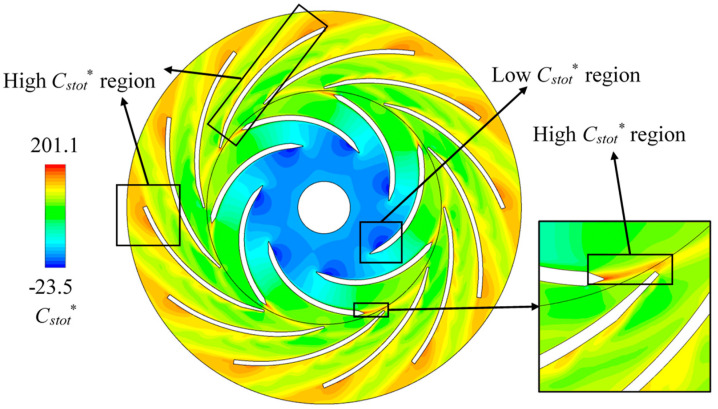
Instantaneous contour of local dissipation coefficient C_stot_^*^ with indications of low and high regions.

The instantaneous C_stot_^*^ contour in the impeller and diffuser is shown in Figure 8. The lowest C_stot_^*^ region is near the impeller blade leading-edge on the suction side. Three mainly high C_stot_^*^ regions can be found at the diffuser outlet, in the impeller trailing-edge wake and near the convex side of diffuser blade. The high C_stot_^*^ regions overlap the low *C_vrel_* regions as shown in the relative velocity contour map. The low C_stot_^*^ regions also overlap the well-behaved flow regions. It revealed that the energy dissipation (transformed to internal energy) is caused by the local undesirable flow regime.

**Figure 9:**
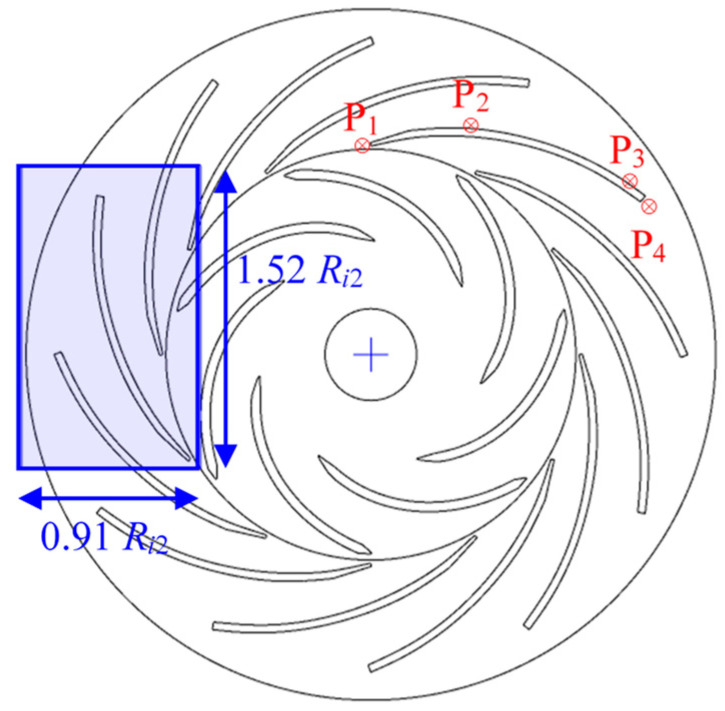
Observation region and monitoring points for the transient pulsation of internal flow characteristics.

However, differences can be found in different blade channels. For example, as shown in the enlarged view in Figure 8, the impeller blade trailing-edge wake may cause high C_stot_^*^ on the diffuser blade leading-edge. On Figure 6, high C_stot_^*^ regions can be obviously found on the leading-edge of 4 of the 12 diffuser blades. The high C_stot_^*^ regions on the convex side of the diffuser blade also only occur in some specific channels. These two high C_stot_^*^ regions seem to be random or rotationally periodic. To understand this phenomenon and its transient change, the internal flow observation is conducted in the region shown in Figure 9 within one impeller revolution. Monitoring points P_1_ to P_4_ are also set in the typical high and low C_stot_^*^ regions as indicated in Figure 9.

**Figure 10:**
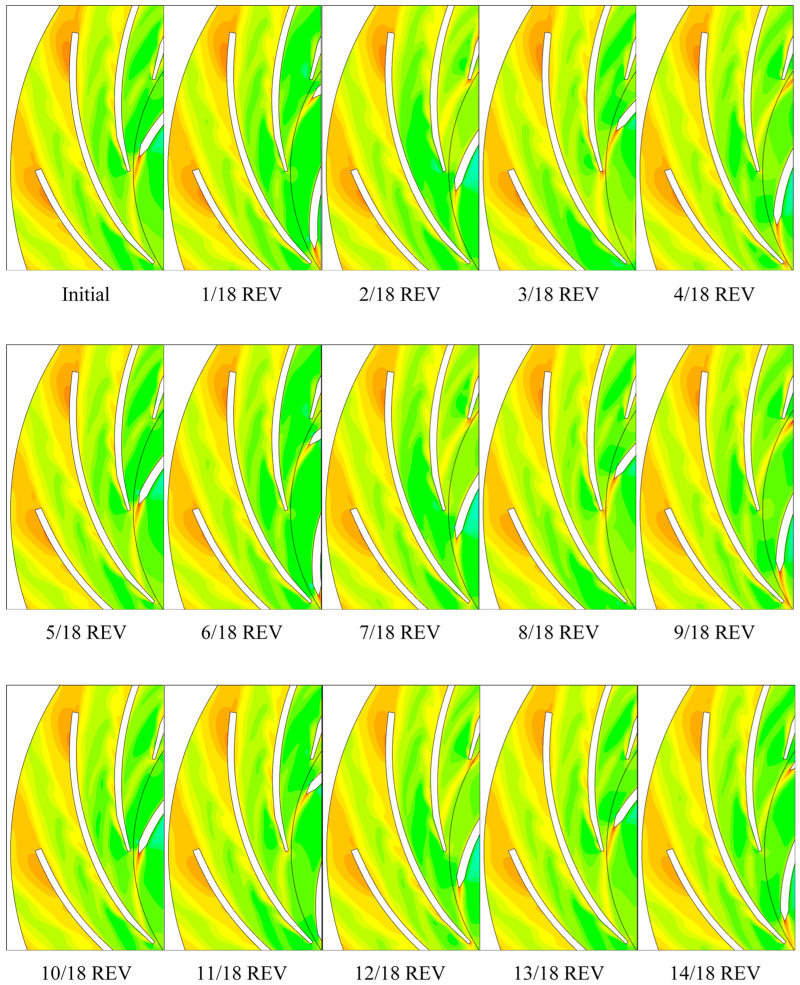

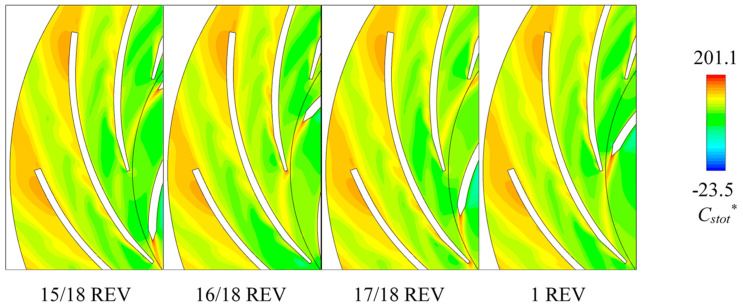
C_stot_^*^ contour pulsation in one impeller revolution. REV: impeller revolution.

Figure 10 shows the C_stot_^*^ contour pulsation in one impeller revolution by plotting sub-maps for each 1/18 revolution. The high C_stot_^*^ region consistently exists on the diffuser blade near outlet which is the stall vortex flow site. However, the high C_stot_^*^ region in the diffuser trailing-edge wake changed periodically. The high C_stot_^*^ regions on the diffuser blade’s convex side also periodically generates and disappears. The high C_stot_^*^ regions form a street from diffuser blade leading-edge to trailing-edge. Another high C_stot_^*^ region occurs periodically on the diffuser leading-edge. Obviously, it is caused by the rotor-stator interaction. When impeller blade trailing-edge wake passes by the diffuser blade leading-edge, high C_stot_^*^ generates. The high C_stot_^*^ region in the impeller blade trailing-edge wake is relatively stable which consistently exists during impeller rotation.

**Figure 11:**
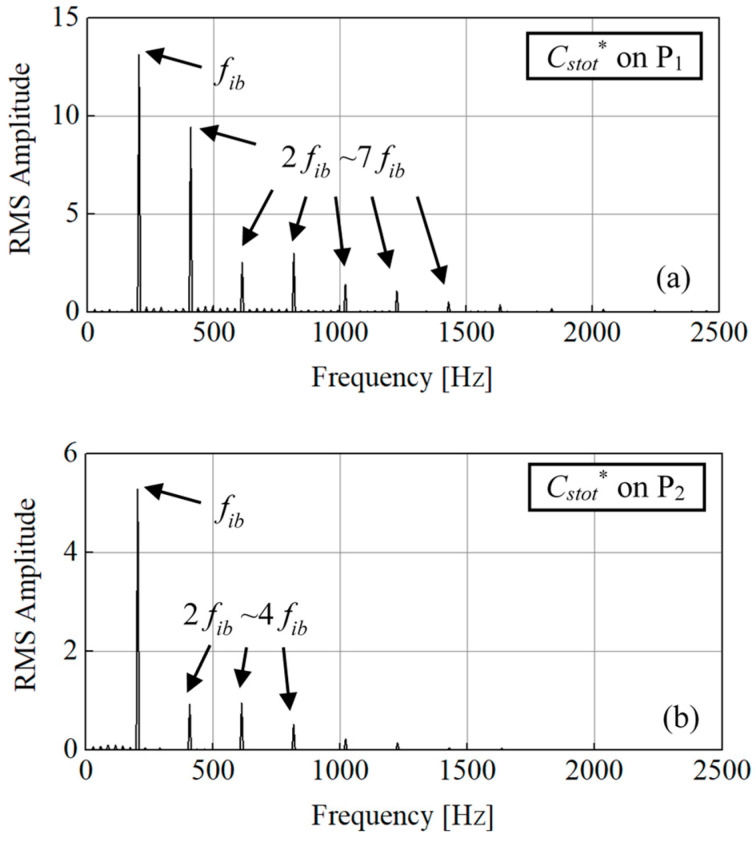

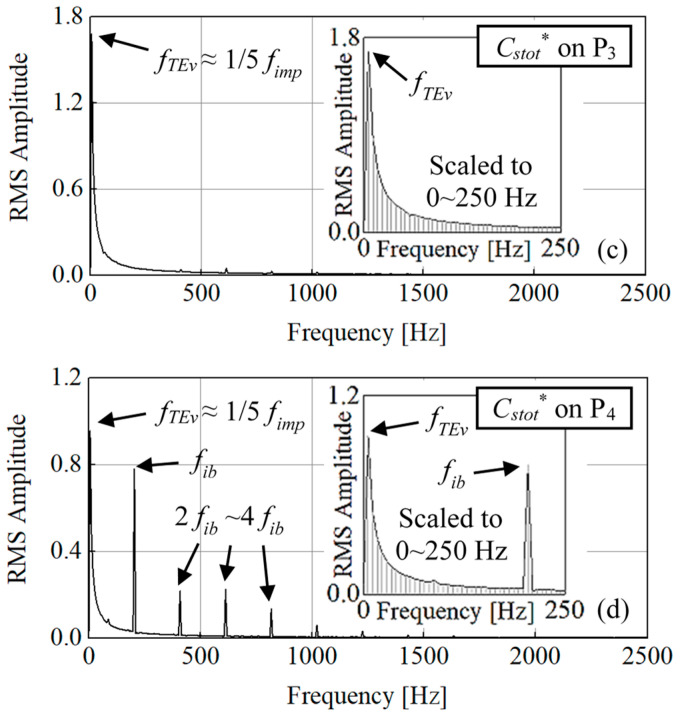
C_stot_^*^ pulsation on P_1_, P_2_, P_3_ and P_4_ within one impeller revolution. (**a**) on P_1_; (**b**) on P_2_; (**c**) on P_3_; (**d**) on P_4_.

Based on the monitoring points P_1_~P_4_ shown in Figure 7, the transient C_stot_^*^ pulsation is analyzed in frequency in Figure 11. According to the rotating speed of 1750 r/min, the impeller frequency *f_imp_* is 29.167 Hz, the impeller blade frequency *f_ib_*=*Z_i_·f_imp_* is 204.167 Hz. Figure 11a is on P_1_ which located on diffuser blade leading-edge and is near the impeller blade trailing-edge. The frequency on P_1_ is mainly dominated by *f_ib_*. The 2-times to 7-times *f_ib_* frequencies are also strong. The C_stot_^*^ on P_1_ seems to be influenced by the rotor-stator interaction. Figure 11b is on P_2_ which located on diffuser blade’s convex side. The frequency that *f_ib_* also dominates and with also 2 to 4 times *f_ib_* peaks. The C_stot_^*^ on P_2_ are also under the rotor-stator interaction. Figure 11c is on P_3_ which is in the stall vortex on diffuser blade trailing-edge near outlet. The dominate frequency is a very-low stall frequency *f_TEv_* which is about 1/5 *f_imp_*. It is mainly and strongly influenced by the stable stalled vortex structure which is similar as in the rotating stall cases [36,37]. Figure 11d is on P_4_ which is in the diffuser blade trailing-edge wake. The frequency on P_4_ becomes complex with both the stall frequency *f_TEv_* and the 1~4 times of impeller blade frequency *f_ib_*. It shows that the C_stot_^*^ in diffuser blade trailing-edge wake is influenced by both the impeller incoming flow and the trailing-edge stall vortex flow.

### 5.3. Turbulence Kinetic Energy Fields

**Figure 12:**
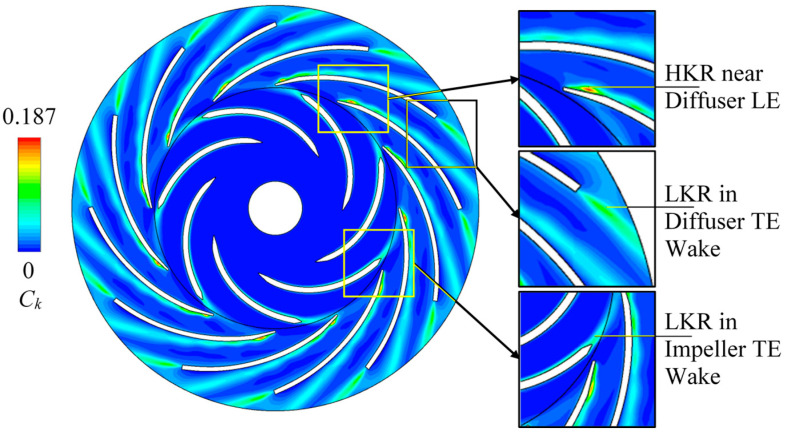
Instantaneous contour of turbulence kinetic energy coefficient *C_k_* with the indications of local low and high regions. HKR: high *k* region; LKR: low *k* region; LE: leading-edge; TE: trailing-edge.

Figure 12 shows the instantaneous contour of turbulence kinetic energy. The turbulence kinetic energy coefficient *C_k_* is defined for analysis:

(16) $$C_{k}=\frac{k}{2gR_{i2}}$$

The high *C_k_* region mainly occurs on the convex side of diffuser blade, in the diffuser blade trailing-edge wake and in the impeller blade trailing-edge wake. These three regions overlap the high C_stot_^*^ regions in Figure 8. However, the stall vortex flow site on diffuser blade near outlet is the low *C_k_* region. It does not accord with the local high C_stot_^*^ characteristic.

In Figure 12, the pattern is not symmetric among the channels. The rotor-stator interaction especially the impeller blade trailing-edge wake influences the *C_k_* values at diffuser inlet.

### 5.4. Flow Induced Noise

**Figure 13:**
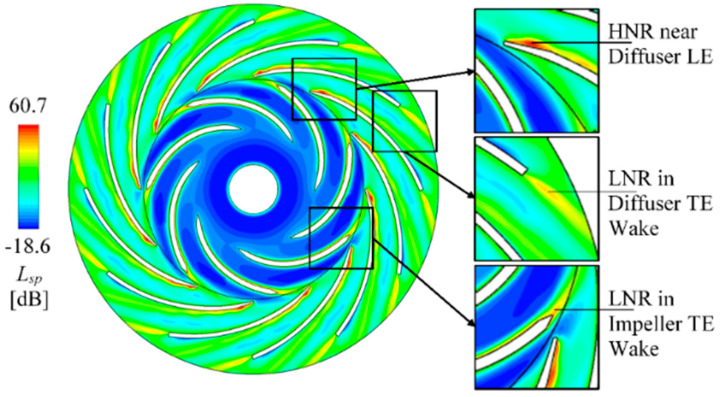
Instantaneous contour of flow-induced noise level *L_sp_* with the indications of local low and high regions. HNR: high noise region; LNR: low noise region; LE: leading-edge; TE: trailing-edge.

The flow induced noise can somehow indicate the extra acoustic energy dissipation. According to Equations (9)–(11), the flow induced noise is strongly related to the turbulence kinetic energy *k* and turbulence eddy dissipation rate *ε*. Therefore, the flow-induced sound power level *L_sp_* is analyzed as the instantaneous contour in Figure 13. The high *L_sp_* regions completely accord with the high *C_k_* regions and are obviously stronger than the surrounded low *L_sp_* sites. To understand the *L_sp_* pulsation during impeller rotation, the *L_sp_* contour is monitored and analyzed within one impeller revolution.

**Figure 14:**
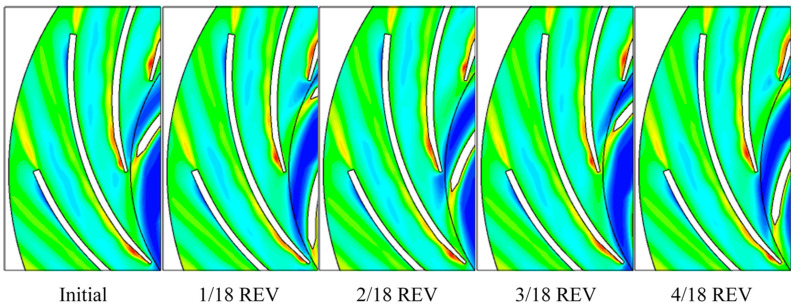

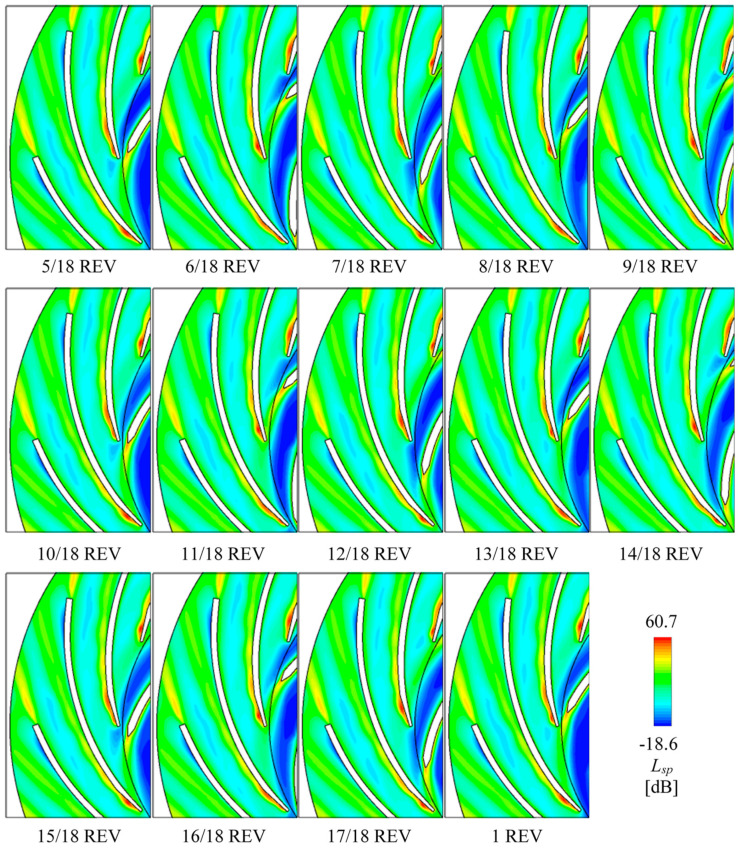
*L_sp_* contour pulsation in one impeller revolution. REV: impeller revolution.

Figure 14 shows the sub-maps for each 1/18 revolution for the transient *L_sp_* fields. The high *L_sp_* regions on diffuser blade are no more a street but are triggered from leading-edge and separate into mid-channel. The diffuser leading-edge high and low *L_sp_* regions are pulsating due to rotor-stator interaction. The stall vortex region on the diffuser blade near outlet is always low in *L_sp_* intensity. The high *L_sp_* regions in the impeller blade trailing-edge wake and in the diffuser blade trailing-edge wake always exist and are relatively stable.

**Figure 15:**
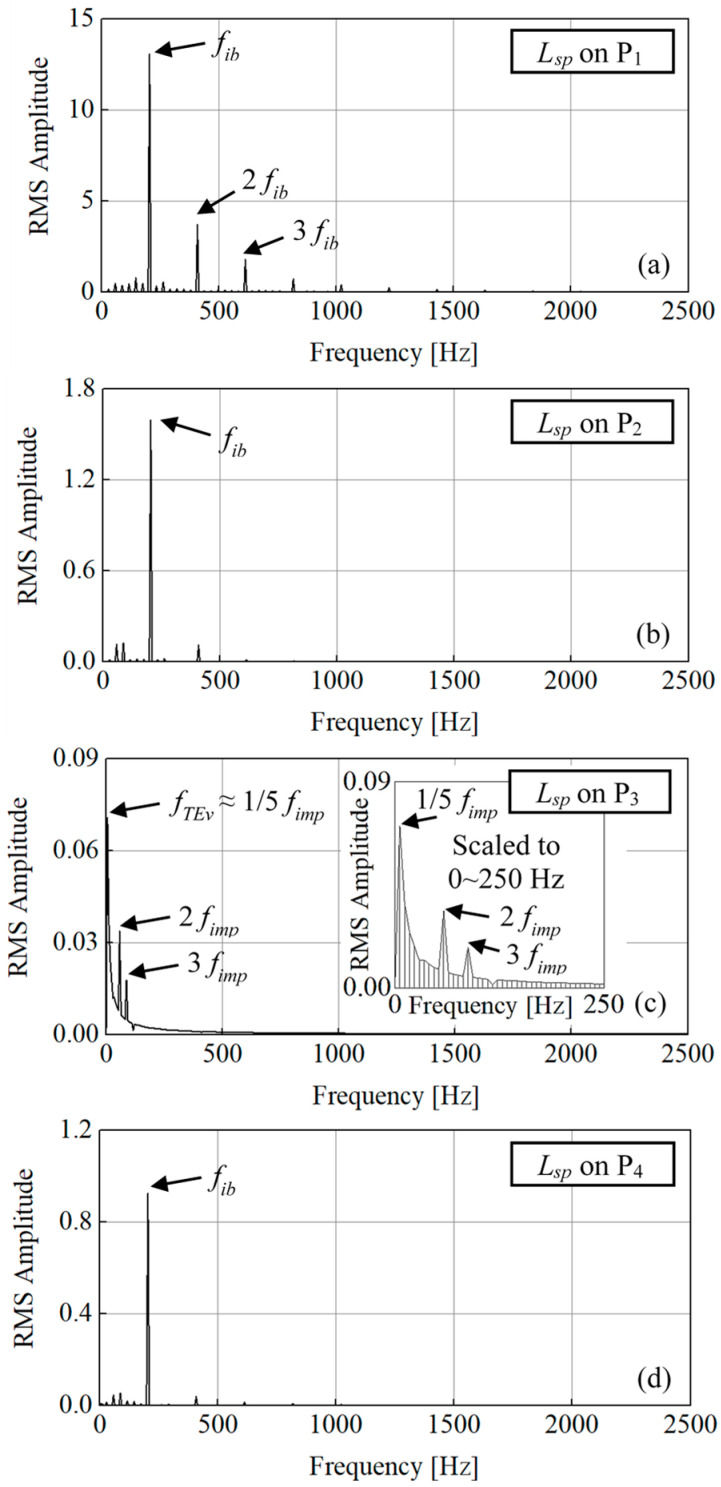
*L_sp_* pulsation on P_1_, P_2_, P_3_ and P_4_ within one impeller revolution. (**a**) on P_1_; (**b**) on P_2_; (**c**) on P_3_; (**d**) on P_4_.

Based on the monitoring points P_1_~P_4_ shown in Figure 7, the transient *L_sp_* pulsation is analyzed in frequency in Figure 15. Figure 15a is on P_1_. The frequency on P_1_ is also mainly dominated by *f_ib_* which is the same as the C_stot_^*^ pulsation. The 2-times and 3-times *f_ib_* frequencies are also strong, which indicate the rotor-stator interaction effect on *L_sp_* on P_1_. Figure 15b is on P_2_. The frequency *f_ib_* is the only dominate frequency greater than other values. The flow-induced noise field on P_2_ is also under the rotor-stator interaction. Figure 15c is on P_3_. The stall frequency *f_TEv_*≈1/5 *f_imp_* is the strongest frequency. The 2 and 3 times *f_imp_* are also obvious as peaks. Both the stall frequency and impeller frequency can be found on the *L_sp_* on P_3_ but the stall frequency dominates. Figure 15d is on P_4_. The *L_sp_* pulsation frequency on P_4_ is not the same as the C_stot_^*^ pulsation frequency. Due to the strong trailing-edge wake, the stall frequency of *L_sp_* caused by trailing-edge stall vortex does not strongly impact the local flow. Thus, frequency *f_TEv_* ≈1/5 *f_imp_* is not found on Figure 15d but the *f_ib_* frequency dominates. The flow induced-noise in the trailing-edge wake is mainly influenced by impeller incoming flow.

## 6. Conclusions

By simulating and analyzing the energy dissipation in centrifugal air pump under rotor-stator interaction, conclusions can be drawn as follows:

(1) The temperature and static entropy patterns can be successfully found by applying the total energy governing equations. The flow energy which transferred to internal energy can be quantitatively known. The flow-induced noise, which might be another energy dissipation source, can be also predicted based on the turbulence flow modeling. It is found strongly relative to the turbulence kinetic energy and dissipation rate.
(2) The high static entropy sites are related to the local low velocity regions. According to the vectors of relative velocity, these low velocity regions have undesirable flow regime. The main high static entropy sites locate in the impeller trailing-edge wake, on the diffuser blade leading-edge and convex side, in the diffuser blade trailing-edge stall vortex near outlet and in the diffuser blade trailing-edge wake. The high noise sites overlap some of the high static entropy sites. These overlapped noisy sites are also due to the local undesirable flow regime. There is an exception as stall vortex near diffuser blade trailing-edge. The flow-induced noise is constantly very low because of the local low turbulence kinetic energy. Accordingly, different flow structures have different energy dissipation mechanisms. In this case, rotor-stator interaction affects both the internal energy and the flow-induced noise. The stalled vortex flow mainly causes the internal energy variation but weak in producing noise.
(3) These high energy dissipation regions perform differently during rotor-stator interaction. Based on the frequency analysis, the transient characteristics of energy dissipation during impeller rotation can be clarified in detail. The flow regime on the diffuser blade leading-edge, on the diffuser blade’s convex side and in the diffuser blade trailing-edge wake are influenced mainly by the impeller frequency or the impeller blade frequency. It shows that the rotor-stator interaction affects the flow regime, energy dissipation and flow-induced noise from diffuser inlet to outlet. The diffuser blade trailing-edge stall vortex is not strongly influenced by rotor-stator interaction. It keeps the stall frequency that is about 1/5 impeller frequency. This stall frequency also affects the diffuser trailing-edge wake region.

Generally, the energy dissipation in the centrifugal air pump is caused by local undesirable flow patterns. These local flow patterns are affected by rotor-stator interaction or stalled flow. Reducing energy dissipation is always the key in turbomachinery’s optimization design. Therefore, understanding and quantifying the local energy dissipation can be helpful for the high-efficiency designs especially in the complex rotor-stator interacting flow cases.

## Figures and Tables

**Table 1:** Geometric parameters of the centrifugal air pump.

Parameter	Value and (Unit)
Impeller blade inlet radius *R_i_*_1_	0.120 (m)
Impeller blade outlet radius *R_i_*_2_	0.210 (m)
Diffuser blade inlet radius *R_d_*_1_	0.222 (m)
Diffuser blade outlet radius *R_d_*_2_	0.332 (m)
Hub arc radius *R_hub_*	0.100 (m)
Shroud arc radius *R_shr_*	0.025 (m)
Impeller inflow radius *R_in_*	0.092 (m)
Cone radius *R_cn_*	0.009 (m)
Height of cone *h_cn_*	0.026 (m)
Height over cone on hub *h_hub_*	0.087 (m)
Height of impeller/diffuser blade *h_b_*	0.020 (m)
Impeller blade number *Z_i_*	7 (-)
Diffuser blade number *Z_d_*	12 (-)

**Table 2:** Performance parameters and medium properties of the centrifugal air pump.

Parameter	Value and (Unit)
Rotational speed *n*	2000 (r/min)
Rotational linear speed at impeller outlet *U_i_*_2_	43.98 (m/s)
Flow rate coefficient *C_φ_*	0.048
Fluid medium dynamic viscosity *μ*	1.83 × 10^−5^ (kg/m·s)
Fluid medium density *ρ*	1.2 (kg/m^3)^
Fluid medium thermal conductivity *λ_t_*	0.0261 (W/m·K)
Fluid specific heat capacity *c_h_*	1004 (J/kg·K)

## Nomenclature

**Latin Letters**			
*A_in_*	impeller inflow area	*Q*	flow rate
*c_h_*	heat capacity	*R_cn_*	Cone radius
*C_k_*	turbulence kinetic energy coefficient	*R*_*d*1_	Diffuser blade inlet radius
*C_r_*	radial component of velocity	*R*_*d*2_	Diffuser blade outlet radius
*C_stot_^*^*	energy dissipation coefficient	*R_hub_*	Hub arc radius
*C_t_*	tangential component of velocity	*R*_*i*1_	Impeller blade inlet radius
*C_vrel_*	relative velocity coefficient	*R*_*i*2_	Impeller blade outlet radius
*C_φ_*	flow rate coefficient	*R_in_*	Impeller inflow radius
*C_ω_*	production term coefficient in SST model	*R_shr_*	Shroud arc radius
*F*_1_	blending function in SST model	*s^*^*	specific entropy
*f_ib_*	impeller blade frequency	*S_ij_*	strain tensor
*f_imp_*	impeller frequency	*S_p_*	spanwise position
*f_TEv_*	diffuser trailing-edge stall frequency	*t*	time
*g*	acceleration of gravity	*T*	temperature
*G_z_*	relative impeller blade channel position	*T_in_*	temperature at inlet boundary
*h_b_*	Height of impeller/diffuser blade	*T_out_*	temperature at outlet boundary
*h_cn_*	Height of cone	*u*	velocity
*h_hub_*	Height over cone on hub	*U*_*i*2_	Rotational linear speed at impeller outlet
*h_sta_*	static enthalpy	*V_c_*	sound speed
*h_tot_*	total enthalpy	*V_in_*	velocity at inlet boundary
*k*	turbulence kinetic energy	*V_out_*	velocity at outlet boundary
l_*k-ω*_	turbulence scale in SST model	*V_rel_*	relative velocity
*L_sp_*	sound power level	*W_A_*	sound power
*M_t_*	specific turbulence kinetic energy	*W_ref_*	reference sound power
*n*	Rotational speed	*x*	coodinate component
*P*	turbulence production term in SST model	*y* ^+^	dimensionless height off-wall
*P*_1_, *P*_2_, *P*_3_, *P*_4_	monitoring point 1 to 4	*Z_d_*	Diffuser blade number
*p_in_*	pressure at inlet boundary	*Z_i_*	Impeller blade number
*p_out_*	pressure at outlet boundary		
**Greek Letters**			
*α_ε_*	model constant in acoustic analogy	*μ_t_*	eddy viscosity
*β_k_*	model constant in SST model	*ρ*	density
*ΔC_stot_*	specific entropy difference coefficient	*σ_k_*	model constant in SST model
*δ_ij_*	Kroneker delta	*σ_ω_*	model constant in SST model
*ε*	eddy dissipation rate	*ϕ*	denotation for arbitrary parameter
*λ_t_*	thermal conductivity		in governing equations
*μ*	dynamic viscosity	*ω*	specific turbulence dissipation rate
**Acronyms**			
CFD	computational fluid dynamics	LNR	low noise region
EXP	experiment	LVR	low *C_vrel_* region
GGI	general grid interface	RANS	Reynolds-averaged Navier-Stokes
HKR	high *k* region	REV	impeller revolution
HNR	high noise region	RMS	root mean square
HVR	high *C_vrel_* region	SS	blade suction side
LE	blade leading-edge	SST	shear stress transport
LKR	low *k* region	TE	blade trailing-edge
